# Dietary nutrient intake and metabolic syndrome risk in Chinese adults: a case–control study

**DOI:** 10.1186/1475-2891-12-106

**Published:** 2013-07-30

**Authors:** Shanshan Bian, Yuxia Gao, Meilin Zhang, Xuan Wang, Weiqiao Liu, Dalong Zhang, Guowei Huang

**Affiliations:** 1 Department of Nutrition and Food Science, School of Public Health, Tianjin Medical University, Tianjin 300070, China; 2 Department of Cardiology, General Hospital of Tianjin Medical University, Tianjin 300052, China; 3 Health Examination Center of Heping District, Tianjin 300070, China

**Keywords:** Metabolic syndrome, Dietary nutrient intake, Niacin, Thiamine, Factor analysis

## Abstract

**Background:**

Because human diets are composed of a wide variety of nutrients that may work synergistically to prevent or promote disease, assessing dietary nutrient intake status may be informative. The purpose of this study was to assess the dietary nutrient intake status of Chinese adults with metabolic syndrome (MetS) and to evaluate its possible role in MetS.

**Methods:**

This case–control study was conducted from March 2010 to January 2011. A total of 123 patients with MetS and 135 controls participated in this study at the Health Examination Center of Heping District in Tianjin, China. Dietary intake was estimated by 24-h dietary recalls. We used principal component factor analysis to derive nutrient groups from 17 major nutrients. We examined the odds ratios and 95% confidence intervals using logistic regression models to test the relationship between tertiles of dietary nutrient pattern and MetS.

**Results:**

There were 4 major dietary nutrient patterns in this study: “vitamin B group”, “protein and lipids”, “vitamin E and minerals”, and “antioxidant vitamins”. After adjustment for potential confounders, the highest tertile of the nutrient pattern factor score for the “vitamin B group” (odds ratio: 0.16; 95% confidence interval: 0.05–0.47) was negatively associated with MetS compared with the lowest tertiles. No relationships were found between other dietary nutrient patterns and MetS.

**Conclusions:**

The “vitamin B group” pattern was inversely associated with MetS in Chinese adults. This finding supports the hypothesis that the “vitamin B group” pattern may have a potentially beneficial effect on the prevention of MetS.

## Background

Metabolic syndrome (MetS) is characterized by a clustering of abdominal obesity, insulin resistance, hypertension, dyslipidemia, and diabetes mellitus, which are considered to contribute to increased incidence of cardiovascular disease and mortality [1,2].

With the economic development of China, the prevalence of MetS has increased significantly. According to the 2002 Chinese National Nutrition and Health Survey, the prevalence of metabolic syndrome in individuals aged ≥18 years was 13.8% based on the Adult Treatment Panel III criteria [3] and has increased strongly over the past few years; some studies have shown that the prevalence of MetS at a regional level is approximately 13.2% to 15.8% [4-6]. With this increase in prevalence, MetS has become a major public health problem [7].

It is known that the aetiology of MetS includes genetic, metabolic, and environmental factors [8], and dietary factors are an important aspect of the environmental factors. Emerging evidence has shown that dietary factors are also associated with MetS [9].

In China, the prevalence of MetS has been rapidly rising, largely reflecting transitions in lifestyle and nutrition [10,11]; intake of grain has decreased significantly, whereas intake of fat has increased dramatically, daily intake of salt is much greater than recommended, and intake of vegetables and fruits is insufficient [3].

In recent years, research studies have examined the effect of specific foods and dietary patterns on MetS [12-16]. Some findings have suggested that intake of fruits and vegetables is associated with a reduced risk of MetS [17-20]. Vegetables and fruits are important contributors of vitamin A, vitamin C, magnesium, and potassium, and they may help support health and wellness and potentially reduce the risk of chronic diseases [13]. MetS has been associated with greater intake of key nutrients. Vitamin D status was assessed by serum 25-hydroxyvitamin D [25(OH)D] concentrations[21]. Data from the Third National Health and Nutrition Examination Survey (NHANESIII) showed an inverse association between 25(OH)D and MetS [22]. Altered vitamin D homeostasis was associated with increased risk of developing MetS [23]. Zinc is involved in the synthesis, storage, and release of insulin. The present study displayed that dietary zinc intake was inversely associated with MetS [24]. Some studies have shown that increased dietary magnesium intake was associated with lower risk of the MetS [25,26].

Because an individual’s diet is composed of a wide variety of foods containing complex combinations of nutrients, research that examines a single nutrient may not adequately account for the complex interactions among all the nutrients contained in certain foods and their cumulative effects on human health. Although there are many studies on single nutrient intake status and MetS, research on the health benefits of dietary nutrient patterns is limited. Meanwhile, we are more interested in vitamins and minerals, which play an important role in the prevention of MetS. Thus, the aim of our study was to investigate the relationship between dietary nutrients, specifically intake of vitamins and minerals in Chinese adults with MetS, which may be more significant for prevention of MetS than research on a single nutrient.

## Methods

### Study population

All individuals were recruited from the Health Examination Center of Heping District in Tianjin, China. In total, 1247 individuals met the criteria for MetS according to the National Cholesterol Education Program- Adult Treatment Panel III (NCEP-ATP III) in the Asian population; 150 patients were randomly selected as the case group, and 123 patients agreed to participate and provided complete information. For the control group without MetS, 150 age- , gender- and residence area–matched subjects with no history of obesity, hyperlipidaemia, hypertension, or diabetes mellitus were selected, and the data from 135 subjects were analysed. The response rate was 86%.

Participants enrolled in the study met the following inclusion criteria: 1) willingness to participate, 2) aged 30–70 years, 3) absence of any clinical disease, 4) not pregnant or lactating, and 5) residing in Tianjin for ≥5 years.

MetS was diagnosed if the patient had 3 or more of the following risk factors, as established by the NCEP-ATP III [27]: 1) waist circumference (WC) ≥90 cm in men or ≥80 cm in women, 2) triglycerides level (TG) ≥1.70 mmol/L, 3) high-density lipoprotein-cholesterol (HDL-c) level <1.03 mmol/L in men or <1.30 mmol/L in women, 4) systolic blood pressure (SBP)/diastolic blood pressure (DBP) ≥130/85 mmHg or current use of antihypertensive medications, and 5) fasting blood glucose (FBG) level ≥5.6 mmol/L or already taking oral hypoglycaemic agents for treatment of type 2 diabetes.

Twenty-seven patients with MetS were excluded from the study because dietary data were not available and questionnaire information was not complete, and 15 controls were excluded because of missing data on physical activity level or any anthropometric measurement and biochemical variable.

Ethical approval for and permission to conduct this study were obtained from the Ethics Committee of Tianjin Medical University, and written informed consent was obtained from all participants.

### Dietary intake

Dietary data were collected using 24-h dietary recalls. Each participant was asked by a trained interviewer to provide the name and amount of all foods consumed. The daily intake of energy and nutrients was averaged over 7 days to estimate usual dietary intake, and the main nutrients of interest were energy, protein, total fat, and cholesterol, and specifically intake of vitamins and minerals. Nutrient intake for each food item consumed was calculated by multiplying the nutrient content listed in the Chinese Food Composition Table [28], and total dietary intake of each nutrient was calculated by adding the intake of that nutrient from each food item consumed. All values obtained for nutrient intake were adjusted for total energy intake using the regression residual method and presented in terms of energy-adjusted values [29].

### Biochemical assessment

Blood samples were collected from each participant while in a seated position after fasting for at least 12 h. Levels of serum TG, total cholesterol (TC), HDL-c, low-density lipoprotein cholesterol (LDL-c), FBG, and uric acid (UA) were measured using a Hitachi 7180 automatic analyser (Hitachi, Japan).

### Anthropometric and lifestyle assessment

Anthropometric measurement and demographic variables were collected from each individual. The blood pressure of each participant was measured twice using a standard mercury sphygmomanometer after he or she had been sitting for 15 min. WC was measured as the narrowest circumference between the bottom of the rib cage and the iliac crest using a tape measure. Height and weight were measured to the nearest 0.5 cm and 0.1 kg with the participant wearing light clothes and no shoes. Body mass index (BMI) was then calculated as weight (kilograms)/height (meters)^2^. Data on demographic variables, including age, gender, educational level, physical activity, and smoking status, were obtained by conducting a questionnaire. The educational level of the participants was reported in years of education. The physical activity level of the participants was recorded during the interview and categorized as 1 of 3 levels at work as recommended by the Chinese Nutrition Society [30]: light (eg, office work, repairing watches and electronics, salesperson, waiter/waitress, teacher), moderate (eg, general activity of students, the productive activities of industry workers, electrician, mechanics), and heavy (eg, the productive activities of non-mechanized agriculture, dance, sports competition, loading or unloading/mine workers). Smoking status was defined as current smoker and non-smoker; current smokers were defined as those who smoked at least one cigarette per day, and non-smokers were former smokers and people who had never smoked a cigarette.

All data are presented as the mean and standard deviation for continuous variables and contingency tables for categorical data and are listed by status of case patients and control subjects. Continuous variables were analysed using the independent-samples t test, and categorical data were examined using the chi-square test. For each subject, the mean of individual nutrients from 24-h dietary recall was used for analysis. To investigate the dietary nutrient intake status in predicting the risk of MetS, we used factor analysis to identify nutrient groups and determine factor loadings.

Principal component factor analysis (PCFA) was used to derive dietary nutrient patterns and to determine factor loadings. Factors were rotated with varimax rotation to maintain uncorrelated factors and enhance interpretability [31]. The number of factors to retain was chosen based on an eigenvalue >1, scree plot test, and factor interpretability. We categorized the tertile cut-offs of dietary nutrient pattern scores based on the factor scores of the controls. Nutrients were found to have rotated factor loadings ≥0.32 [32]. We estimated the odds ratio (OR) and 95% confidence interval (CI) for each tertile using logistic regression models. These analyses were adjusted for various variables in 2 models. Model 1 was adjusted for age, gender (male/female), education level, smoking status (non-smoker/current smoker), and physical activity level (light, moderate, and heavy). Model 2 included an additional adjustment for BMI, which was included as a covariate to isolate the independent effects of central adiposity as measured by WC.

All statistical analyses were performed using Statistical Package for the Social Sciences software version 16.0 (SPSS Inc., Chicago, IL, USA). In this study, a P-value <0.05 was considered significant.

## Results

A total of 258 subjects enrolled in the study. Clinical characteristics, lifestyle factors, and dietary nutrient intake status are shown in Table 1. The patients with MetS had significantly higher blood pressure, BMI, and WC and higher levels of TG, TC, LDL-c, FBG, and UA but lower HDL-c levels than those in the control group. The patients with MetS were more likely to be smokers and had a lower education level. In regard to dietary nutrient intake status, intake of energy, total fat, cholesterol, and sodium were significantly higher and intake of vitamin E and magnesium were relatively deficient in the patients with MetS compared with the control group. There were no significant differences in gender, age, and physical activity level between the 2 groups.

**Table 1:** Basic characteristics and dietary nutrient intake of case and control subjects

**Variable**	**Case subjects**	**Control subjects**	
	**N = 123**	**N = 135**	
	**Frequency (%)**	**Frequency (%)**	***P*-value**^**1**^
**Gender**			0.90
Female	51 (41.4)	57 (42.2)	
Male	72 (58.5)	78 (57.8)	
**Physical activity level**			0.53
Heavy	15 (12.2)	21 (15.6)	
Moderate	50 (40.7)	59 (43.7)	
Light	58 (47.2)	55 (40.7)	
**Smoking status**			0.03
Current smoker	52 (42.3)	40 (29.6)	
Non-smoker	71 (57.7)	95 (70.4)	
	**Mean ± Std Dev**	**Mean ± Std Dev**	***P*-value**^**2**^
Education level, years	14.4 (2.0)	15.3 (1.5)	<0.0001
Age, years	54.0 (10.4)	53.7 (9.7)	0.89
SBP, mmHg	144 (16.0)	118 (14.4)	<0.0001
DBP, mmHg	87 (9.3)	75 (9.5)	<0.0001
BMI, kg/m^2^	28.2 (3.0)	23.3 (2.7)	<0.0001
WC, cm	88.0 (9.6)	81.2 (8.2)	<0.0001
TC, mmol/l	5.4 (1.1)	4.8 (0.8)	<0.0001
TG, mmol/l	2.7 (1.8)	1.1 (0.9)	<0.0001
HDL-c, mmol/l	1.3 (0.2)	1.4 (0.3)	<0.0001
LDL-c, mmol/l	3.2 (0.8)	2.7 (0.7)	<0.0001
FBG, mmol/l	6.9 (1.8)	5.5 (1.5)	<0.0001
Uric acid, μmol/l	341.1 (52.4)	306.7 (58.1)	<0.0001
Nutrients			
Energy, kcal	2198.4 (596.5)	1966.0 (305.3)	<0.0001
Protein, g	70.4 (20.5)	68.9 (14.5)	0.49
Total fat, g	81.5 (26.4)	71.8 (17.0)	<0.05
Cholesterol, mg	498.7 (267.3)	430.7 (215.5)	<0.05
Vitamin A , μg RE	600.3 (155.5)	629.4(360.0)	0.55
Thiamine, mg	0.9 (0.3)	1.9 (1.1)	0.32
Riboflavin, mg	0.9 (0.3)	1.6 (0.8)	0.34
Niacin, mg	15.0 (7.1)	15.3 (9.9)	0.83
Vitamin C, mg	101.9 (41.4)	106.0 (46.5)	0.58
Vitamin E, mg	27.5 (8.7)	29.9 (8.7)	<0.05
Calcium, mg	457.5 (207.3)	489.1 (178.1)	0.19
Magnesium, mg	303.3 (78.6)	332.3 (77.8)	<0.05
Iron, mg	23.0 (7.3)	23.3 (10.7)	0.82
Zinc, mg	12.5 (3.0)	11.9 (2.2)	0.10
Selenium, μg	58.9 (23.8)	57.9 (17.5)	0.72
Potassium, mg	1813.6 (377.3)	1836.0 (513.1)	0.69
Sodium, mg	3698.3 (868.2)	3377.6 (685.5)	<0.0001
Copper, mg	2.0 (1.6)	1.9 (0.6)	0.41

^1^ P-value for chi-square test

^2^ P-value for independent-samples t test

Dietary nutrients and factor loading scores are presented in Table 2. Factor 1, identified as the “vitamin B group” pattern, and was characterized by high levels of consumption of thiamine, riboflavin, and niacin. Factor 2 was typified by greater levels of consumption of protein, total fat, selenium, and cholesterol and was named the “protein and lipids” pattern. Factor 3 had the greatest absolute loading of vitamin E, iron, potassium, and magnesium and was identified as the “vitamin E and minerals” pattern. Factor 4 was typified by greater levels of consumption of vitamin A, vitamin C, and calcium and was named the “antioxidant vitamins” pattern. These 4 patterns explained 52.65% of the variance in dietary nutrient consumption (17.84% for factor 1, 13.98% for factor 2, 13.11% for factor 3, and 7.72% for factor 4).

**Table 2:** Factor loading matrix and explained variance for nutrient patterns identified by factor analysis

**Nutrient**	**Factor 1**	**Factor 2**	**Factor 3**	**Factor 4**
Protein	0.18	**0.86**	−0.05	0.06
Total fat	0.13	**0.69**	0.01	0.12
Cholesterol	−0.04	**0.66**	0.05	0.05
Vitamin A	0.18	0.10	0.10	**0.37**
Thiamine	**0.97**	0.06	−0.00	−0.00
Riboflavin	**0.97**	0.07	0.00	0.01
Niacin	**0.88**	0.05	−0.05	−0.03
Vitamin C	0.02	−0.21	−0.04	**0.73**
Vitamin E	0.00	−0.09	**0.82**	0.06
Calcium	−0.08	0.30	−0.04	**0.68**
Magnesium	−0.06	0.05	**0.74**	0.05
Iron	0.02	0.08	**0.68**	−0.09
Zinc	0.11	0.00	−0.05	−0.07
Selenium	−0.06	**0.77**	−0.10	−0.20
Potassium	−0.00	−0.08	**0.76**	−0.08
Sodium	−0.06	0.10	−0.04	0.04
Copper	−0.04	0.15	0.08	0.29
Proportion of explained variance (%)	17.84	13.98	13.11	7.72
Cumulative explained variance (%)	17.84	31.82	44.93	52.65

NOTE: Estimates from a PCFA done on 17 nutrients. Loadings ≥0.32 are shown in boldface.

We examined the adjusted ORs (95% CIs) of tertiles of dietary nutrient patterns and MetS (Table 3). The highest tertile of the vitamin B group pattern (OR: 0.16; 95% CI: 0.05–0.47; P-value = 0.001) was associated with the risk of developing MetS after adjustment for age, sex, education level, smoking status, physical activity, and BMI. However, the “protein and lipids”, “vitamin E and minerals”, and “antioxidant vitamins” patterns were not significantly associated with the risk of developing MetS.

**Table 3:** OR of MetS and corresponding 95%CIs on tertiles of factor scores from a PCFA

**Nutrient pattern**	**Tertiles of dietary nutrient pattern factor score**			
	**Low**	**Middle**	**High**	***P *for trend**
Vitamin B group				
Model 1	1	0.47 (0.24, 0.95)	0.38 (0.18, 0.76)	0.007
Model 2	1	0.21 (0.08, 0.57)	0.16 (0.05, 0.47)	0.001
Protein and lipids				
Model 1	1	0.37 (0.19,0.75)	0.98 (0.49, 1.96)	0.96
Model 2	1	0.43 (0.16, 1.14)	1.20 (0.46, 3.14)	0.71
Vitamin E and minerals				
Model 1	1	0.90 (0.45, 1.79)	0.73 (0.37, 1.44)	0.36
Model 2	1	1.64 (0.63, 4.25)	0.73 (0.29, 1.86)	0.51
Antioxidant vitamins				
Model 1	1	3.24 (1.56, 6.73)	1.30 (0.65, 2.63)	0.46
Model 2	1	2.89 (1.04, 7.96)	2.22 (0.82, 5.94)	0.11

Model 1 was adjusted for age, sex, education level, smoking status and physical activity level.

Model 2 was adjusted for all the aforementioned factors as well as BMI.

## Discussion

This case–control study investigated the relationship between dietary nutrient intake status and MetS. Our results suggested that the “vitamin B group” pattern was negatively associated with the prevalence and incidence of MetS.

The “vitamin B group” examined here included thiamine (vitamin B_1_), riboflavin (vitamin B_2_), and niacin (vitamin B_5_). Both thiamine and riboflavin are water-soluble vitamins that cannot be synthesized by the human body and must be consumed daily at an adequate level [33,34].

Our hypothesis was that the “vitamin B group” pattern was negatively associated with the risk of MetS. Several studies of dietary nutrients may partly support our hypothesis. Firstly, a recent study found that thiamine supplementation may affect metabolic abnormalities in Otsuka Long-Evans Tokushima Fatty (OLETF) rats, which are bred to exhibit obesity and metabolic disorders similar to those experienced by humans with MetS [35]. Thiamine is necessary for many metabolic processes, including endocrine and exocrine functioning of pancreatic β cells. Thiamine deficiency leaded to impaired insulin synthesis and secretion and ultimately the development of insulin resistance (IR) and macrovascular disease [36,37]. Several experimental and clinical studies have used thiamine supplementation to treat various genetic disorders linked to metabolic pathway dysfunction, including MetS [33,38,39]. Dietary cereals (especially breakfast cereals) appeared to be more useful in the maintenance of an adequate thiamine status [40]. Secondly, several studies have shown that intake of niacin, a broad-spectrum lipid-modulating agent, can have significantly beneficial effects on atherogenic dyslipidemia by reducing serum TG and LDL-c levels and increasing the HDL-c level [41,42]. In addition, several studies have found that niacin supplementation has beneficial effects on lipid levels in patients with MetS, diabetes, and atherosclerosis, and the pharmacological level of niacin required can treat lipid disorders and cardiovascular disease (CVD) [43]. A Korean study concluded that adequate daily intake of niacin reduced the risk of MetS, whereas inadequate intake increased the risk [44]. Riboflavin is unique among the water-soluble vitamins in that cereals, meat, and fish are good sources of riboflavin, and certain fruits and vegetables, especially dark-green vegetables, contain reasonably high concentrations [45]. Daily consumption of breakfast cereal with milk would be expected to maintain an adequate intake of riboflavin [46-48]. Current interest is focused on the role of riboflavin in determining circulating concentrations of homocysteine, a risk factor for CVD [45]. Riboflavin is required as a correlative factor, and enhanced riboflavin status results in a marked lowering of homocysteine levels, which may be of benefit in treatment of CVD [49]. A relational study shown that riboflavin deficiency may exert some of its effects by reducing the metabolism of other B vitamins [45], and long-term supplementation with riboflavin and pyridoxine HCl (vitamin B_6_ hydrochloride) improved erythrocyte vitamin B_2_ and B_6_ levels [50]. The complex interactions among these nutrients might be related to MetS, which should be clarified in a future study. Our findings suggest that the “vitamin B group” pattern as a whole may be a more important factor that influences the prevalence of MetS than a single nutrient. These nutrients, together with MetS, may cooperatively mediate the beneficial association with the “vitamin B group” pattern. Vitamin B group are rich in nuts, millet, beans foods, and certain fruits (such as tangerines, peaches, and grapes) [28,45]. Although different vegetables have different B vitamin contents, dark-green vegetables, peppers, and cruciferous vegetables are generally good sources of B vitamins [51]. The priority of the modern diet should be low-processed cereal products that contain high levels of B vitamins. Czaja et al. showed that the richest source of thiamine and niacin is wild rice [52]. Further, a long-term study is required to clarify the association between the vitamin B group pattern and MetS in the future.

Oxidative stress is invariably associated with MetS. Although epidemiological studies have demonstrated that vitamin C and vitamin E decrease the incidence of coronary heart disease, clinical trials have failed to support the beneficial effect of these antioxidants [53]. Meanwhile, as a part of vitamin A, retinol + retinyl esters had a positive relationship with IR, UA, and MetS [54]. Samara et al. showed that calcium levels were positively related to TG and negatively to HDL-c in women [55]. Our findings demonstrated that there was no negative correlation between the antioxidant vitamins group and MetS, but this relationship must be confirmed in a future study.

To the best of our knowledge, this was the first study to examine dietary nutrient patterns on the risk of MetS. However, this study has several limitations. First, the sample size was relatively small and the study population does not reflect the general population, so our results may not be extended to other populations. Second, energy and nutrient intake in this study were based on a single 24-h dietary recall, which are frequently used in dietary assessment and intake for 7 days in our study was an estimator of long-term usual intake. Although we adjusted for a number of potential confounding factors, we still cannot avoid the possibility of recall bias, unresponsive bias, and other unknown confounding factors, which might influence the result of risk factor analysis. Therefore, a prospective study should be undertaken to confirm the existence of a relationship between dietary nutrient intake and MetS. Further study is required to clarify the possible mechanism underlying the relationship between the vitamin B group pattern and MetS.

## Conclusions

In conclusion, our study showed that the “vitamin B group” pattern was negatively associated with the risk of MetS in Chinese adults. Our findings suggest that intake of B vitamins as a whole might be a simple and effective way to prevent or slow the development of MetS.

## Abbreviations

MetS: Metabolic syndrome; NCEP-ATP III: National cholesterol education program- adult treatment panel III; BP: Blood pressure; SBP: Systolic blood pressure; DBP: Diastolic blood pressure; FBG: Fasting blood glucose; HDL-c: High-density lipoprotein cholesterol; LDL-c: Low-density lipoprotein cholesterol; TG: Triglyceride; WC: Waist circumference; UA: Uric acid; BMI: Body mass index; PCFA: Principal component factor analysis; OLETF: Otsuka long-evans tokushima fatty; IR: Insulin resistance; CVD: Cardiovascular disease; pyridoxine HCl: vitamin B_6_ hydrochloride.

## Competing interests

The authors declare that they have no competing interests.

## Authors’ contributions

The authors’ contributions to manuscript were as follows: SB and YG: acquired the data, analysed and interpreted the data, performed statistical analysis, and drafted the manuscript. MZ: acquired the data and performed statistical analysis. XW and WL: acquired the data and analysed and interpreted the data. DZ: acquired the data and performed statistical analysis. GH: conceived and designed the research, analysed and interpreted the data, handled funding and supervision, drafted the manuscript, and critically revised the manuscript for intellectual content. All authors read and approve the final manuscript.
